# Mesenchymal Stem Cells Activate the MEK/ERK Signaling Pathway and Enhance DNA Methylation via DNMT1 in PBMC from Systemic Lupus Erythematosus

**DOI:** 10.1155/2020/4174082

**Published:** 2020-11-17

**Authors:** Hui Xiong, Zhixuan Guo, Zengqi Tang, Xuechen Ai, Qing Qi, Xiuting Liu, Danqi Huang, Zhaofeng Li, Suyun Ji, Qing Guo

**Affiliations:** ^1^Department of Dermatology, Sun Yat-sen Memorial Hospital, Sun Yat-sen University, Guangzhou, Guangdong 510120, China; ^2^Guangdong Provincial Key Laboratory of Malignant Tumor Epigenetics and Gene Regulation, Medical Research Center, Sun Yat-sen Memorial Hospital, Sun Yat-Sen University, Guangzhou, Guangdong 510120, China; ^3^Department of Dermatology, The Eighth Affiliated Hospital, Sun Yat-sen University, Shenzhen, Guangdong 518033, China; ^4^Department of Orthopedics, Sun Yat-sen Memorial Hospital, Sun Yat-sen University, Guangzhou, Guangdong 510120, China; ^5^Department of Dermatology, Dermatology Hospital of Southern Medical University, Guangdong Provincial Dermatology Hospital, Guangzhou, Guangdong 510120, China

## Abstract

The defective MEK/ERK signaling pathway and downstream hypomethylation pattern of lymphocytes are crucial for the pathogenesis of systemic lupus erythematosus (SLE). However, the role that the mesenchymal stem cells play in the MEK/ERK signaling pathway and DNA methylation of peripheral blood mononuclear cells (PBMC) from SLE patients remains unknown. In this study, we found that the MEK/ERK signaling pathway of PBMC from SLE patients was activated after the coculture with bone marrow-derived mesenchymal stem cells (BM-MSC) compared with that from the control group. In addition, the expression level of DNA methyltransferase 1 (DNMT1) increased while the levels of CD70, integrin, alpha L (ITGAL), selectin-l, and IL-13 were reduced in PBMC from SLE patients. However, no obvious effect of BM-MSC on PBMC from healthy controls was observed. These findings revealed that BM-MSC might downregulate the expression of methylation-sensitive genes and then suppress the autoactivated PBMC via the MEK/ERK signaling pathway. And it may be one of the mechanisms that BM-MSC ameliorated SLE.

## 1. Introduction

Characterized by the deposition of autoantibodies and immune complexes resulting in inflammation of multiple organs, systemic lupus erythematosus (SLE) is a refractory autoimmune disease involved in 2.2 to 23.1 out of 100000 per year globally [1]. It is widely accepted that the excessive activation of lymphocytes contributes to the pathogenesis of SLE [2]. The epigenetics including DNA methylation, histone modification, and noncoding RNA has been considered extremely important in the pathogenesis of SLE in recent decades [3]. Indeed, the epigenetic regulation especially the DNA hypomethylation of T cells and the defective extracellular signal-regulated kinase (ERK) pathway has been extensively studied in SLE and brought great attention [4, 5]. Therefore, activating the ERK signaling pathway and restoring the hypermethylation of T cells may be a potential treatment for lupus.

Bone marrow mesenchymal stem cells (BM-MSC) are multiple potent stem cells that can differentiate into multiple cell lines [6]. The immunomodulatory function of BM-MSC has been well studied in various diseases. Thus, the transplantation of BM-MSC has been regarded as a new therapy for inflammatory diseases including lupus either alone or in combination with other drugs [7]. To date, the transplantation of MSC has been proven to be an efficient and safe treatment due to its powerful immunoregulation and lower immunogenicity [8, 9]. BM-MSC exert such beneficial influence on multiple aspects. BM-MSC can reduce the levels of proinflammatory cytokines, suppress the proliferation of T cells and the expansion of Th17 and Tfh cells, promote the proliferation of Treg cells [10], and so on [11, 12]. However, the underlying mechanism remains unclear.

The MEK/ERK signaling pathway is an important axis of regulating DNA methyltransferase [13]. The activation of the ERK pathway can upregulate DNA methyltransferase 1 (DNMT1) (a member of the cytosine methylate family that can catalyze the addition of methylation marks to genomic DNA) [14], through which the DNA methylation is modified. Several studies have discovered the defective ERK pathway [15] and overexpression of methylation-sensitive genes [16] (including CD70, ITGAL, perforin, selectin-l, IL-4, and IL-13) of lymphocytes from SLE patients compared with the healthy control [17, 18].

In this study, we carried out, for the first time, a study to evaluate the MEK/ERK signaling pathway of PBMC isolated from SLE patients after the coculture with BM-MSC. In addition, the effect of BM-MSC on the expression of DNMT1 and DNA hypomethylation of PBMC from SLE patients was also investigated.

## 2. Materials and Methods

### 2.1. Patients, Controls, and Blood Samples

SLE patients were recruited from the dermatology inpatient department in Sun Yat-sen Memorial Hospital, Sun Yat-sen University (Guangzhou, China). SLE was diagnosed according to the 1997 revised American Rheumatism Association criteria. All patients were diagnosed with SLE for the first time or had an SLE disease activity index (SLEDAI) equal to or above 6. The sex- and age-matched healthy volunteers were recruited as the control group. 20 ml EDTA anticoagulated venous peripheral blood was obtained from SLE patients and control subjects. The study was approved by the Clinical Research Ethics Committee of Sun Yat-sen University (permit no. SYSEC-KY-KS-2015-021). The healthy volunteers and the patients had read and signed the informed consent.

### 2.2. BM-MSC Culture and Differentiation

BM-MSC in the third generation from healthy volunteers were purchased from the Stem Cell Therapy Center of Sun Yat-sen Memorial Hospital. MSC were cultured with additional 2 generations and then cultured in the low-glucose Dulbecco modified Eagle's medium LG-DMEM (C11885500, Gibco, USA) supplemented with 10% FBS (10099-141, Gibco, USA), 100 U/ml penicillin (15140, Gibco, USA), 100 *μ*g/ml streptomycin (15140, Gibco, USA), 0.4 mM L-glutamine (25030, Gibco, USA), and 10 ng/ml recombinant human FGF-basic (100-18B, PeproTech, USA). For osteogenic induction, MSC were seeded in a 12-well plate (0.6 × 10^5^ per well) with complete medium. The medium of MSC was changed into bone induction medium (adding 0.1 *μ*M dexamethasone (D4902, Sigma-Aldrich, USA), 10 mmol/l *β*-glycerophosphate (G9422, Sigma-Aldrich, USA), and 50 *μ*mol/l ascorbate (A4403, Sigma-Aldrich, USA) in the complete medium) at 80% confluence and then cultured for additional 4 weeks. Then, osteogenic differentiated MSC were stained with 1% Alizarin red (G1452, Solarbio, China). For adipogenic induction, we changed the complete medium into adipogenic inducing medium (adding 1 *μ*M dexamethasone, 10 *μ*g/ml insulin (I8830, Solarbio, China), and 0.5 mmol/l IBMX (I8450, Solarbio, China) in the complete medium when the cell is at 100% confluence) and cultured it for 3 weeks. Then, adipogenic differentiated MSC were verified by Oil Red O (G1262, Solarbio, China) staining and counterstained with hematoxylin (H8070, Solarbio, China).

### 2.3. Flow Cytometry

MSC were stained with FITC-conjugated anti-CD105 (#561443), CD44 (#555478), and CD45 (#555482) and PE-conjugated CD90 (#555596), CD73 (#550257), CD29 (#555443), and CD34 (#555822) monoclonal antibodies (Becton Dickinson, USA) or with appropriate control antibodies and analyzed with a flow cytometer (BD FACSVerse, USA).

### 2.4. PBMC Isolation

Peripheral blood mononuclear cells (PBMC) were isolated from EDTA anticoagulated by Ficoll-Paque density gradient centrifugation using the lymphocyte separation medium (LTS1077, TBD, China). Then, cells were washed twice with PBS and cultured in Roswell Park Memorial Institute- (RPMI-) 1640 medium (C11875500, Gibco, USA) supplemented with 10% FBS, 100 U/ml penicillin, 100 *μ*g/ml streptomycin, and 0.4 mM L-glutamine. Cells were maintained in a humidified incubator at 37°C with 5% CO_2_.

### 2.5. Coculture Assay

After the BM-MSC were cultured in the 5th generation, trypsin-EDTA solution was added to prepare the single-cell suspension. Then, the single-cell suspension of BM-MSC was seeded in the 24-well plate (6.7 × 10^4^ per well) with LG-DMEM complete medium before the coculture. 12 hours later, the MSC grew adhering to the well plate. Then, the DMEM was removed and the freshly isolated PBMC (1 × 10^6^cells per well) were seeded with RPMI-1640 complete medium. Since the PBMC were suspension cells while the BM-MSC were adherent cells, we could observe that the BM-MSC stick to the bottom of the well plate and the PBMC were suspended in the medium. As a result, both of them were in contact with each other. The direct contact coculture system was established (ratio of PBMC : BM‐MSC = 15 : 1). For the control group, PBMC were seeded alone in the 24-well plate. The cells from both the coculture and control groups were stimulated [19] with 10 ng/ml IL-2 (200-02, PeproTech, USA), 1 *μ*g/ml anti-CD28 (555336, BD, USA), and 0.2 *μ*g/ml anti-CD3 (555725, BD, USA). After the coculture with BM-MSC, PBMC were isolated again from the mixed cells by Ficoll-Paque density gradient centrifugation using the lymphocyte separation medium.

### 2.6. Western Blot Analysis

PBMC were washed once with PBS and lysed with RIPA buffer (P0013B, Beyotime, China) with protease (CW2200S) and phosphatase inhibitors (CW2383S, CWBIO, China). Antibodies against p-MEK (#9154, 45 kDa), MEK (#8727, 45 kDa), p-ERK (#4370, 44, 42 kDa), ERK (#4695, 44, 42 kDa) (1 : 1000 dilution, Cell Signaling Technology, USA), DNMT1 (1 : 500 dilution, ab18453, 183 kDa, Abcam, USA), and GAPDH (10494-1-AP, 36 kDa, Proteintech, USA) were used for immunoblotting according to the manufacturers' protocols.

### 2.7. Real-Time PCR Analysis

Total RNA of PBMC was isolated with RNAiso Plus (9108, Takara, Japan) followed by reverse transcription (RT) with the PrimeScript RT Master Mix (RR036A, Takara, Japan). The mRNA levels encoding DNMT1, CD70, ITGAL, perforin, selectin-l, IL-4, and IL-13 were assessed by real-time PCR using the TB Green system (RR820A, Takara, Japan) following the manufacturer's protocol. The relative expression of the target mRNA was normalized to the expression of *β*-actin mRNA. The primers were designed spanning exons to avoid genomic DNA amplification, as described in Table 1.

### 2.8. Statistical Analysis

Data were presented as the mean ± SD. Statistical comparisons were performed using one-way ANOVA and determined by the Tukey test. All analyses were performed with the GraphPad Prism 8.0 software. The statistical significance level was set as ^∗^*P* < 0.05, ^∗∗^*P* < 0.01, and ^∗∗∗^*P* < 0.001.

## 3. Results

### 3.1. Identity of Bone Marrow Mesenchymal Stem Cells

**Figure 1:**
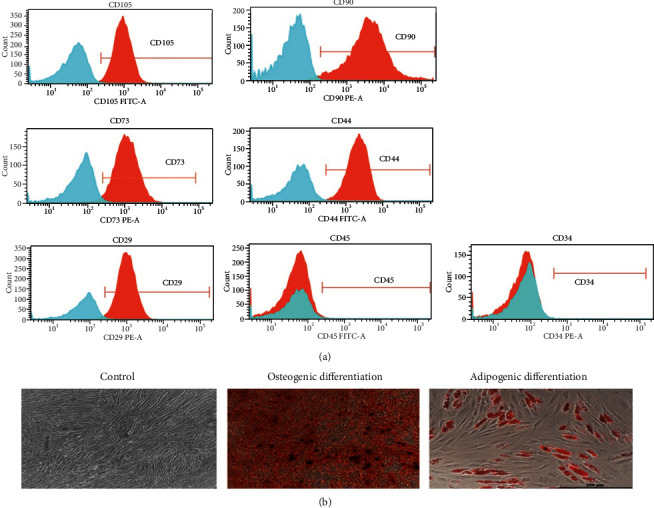
Identification of bone marrow mesenchymal stem cells. (a) BM-MSC express specific surface markers. After being cultured on the 5th generation, BM-MSC from healthy volunteers were stained with FITC-conjugated anti-CD105, CD44, and CD45 and PE-conjugated CD90, CD73, CD29, and CD34 monoclonal antibodies and appropriate controls. (b) The osteogenic and adipogenic differentiation of BM-MSC. The BM-MSC of the same generation were cultured with normal complete medium (adding b-FGF to help keep multipotent), osteogenic medium, or adipogenic medium. MSC differentiated into osteocytes characterized by Alizarin red staining (middle, 40x) and adipocytes characterized by lipid vacuoles (right, 100x).

To investigate the immunomodulation mechanism of the human MSC, we firstly isolated the MSC from healthy volunteers. According to the previous reports, BM-MSC express specific surface markers including CD105, CD90, CD73, CD44, and CD29. The MSC we cultured were characteristically positive for CD105, CD90, CD73, CD44, and CD29 by flow cytometric analysis and negative for hematopoietic antigens such as CD45 and CD34 (Figure 1(a)), which was in agreement with previous studies. Furthermore, it has been agreed that MSC has multiple differentiation potential. Here, human MSC successfully differentiated into osteoblasts and adipocytes (Figure 1(b)), both of which were defined by the abundant lipid vacuoles or the deposition of a calcium-rich mineralization, respectively.

### 3.2. MEK/ERK Pathway of SLE PBMC Is Activated, and the Level of DNMT1 Increases after the Coculture with MSC

**Figure 2:**
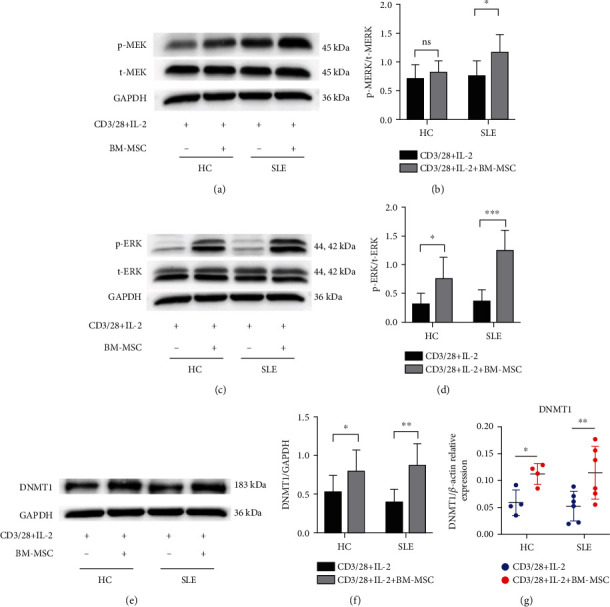
MEK/ERK pathway of SLE PBMC is activated, and DNMT1 increases after the coculture with MSC. PBMC were isolated from healthy controls and lupus patients. After being stimulated with IL-2, anti-CD28, anti-CD3, both of them were divided into the single-culture group and coculture group (PBMC : BM‐MSC = 15 : 1). (a, c) The expressions of p-MEK, t-MEK, p-ERK, and t-ERK of PBMC were assessed by western blot analysis after 3 days. (b, d) The densitometry analysis of the results (a, c) is shown as the ratio of phosphorylated protein and total protein. (e–g) The protein and mRNA of DNMT1 of PBMC were determined by real-time PCR and western blot, respectively, after 4 days. The data of (a)–(d) are presented as the mean ± SD of 8 healthy volunteers and 8 patients. The data of (e)–(g) are represented as the mean ± SD of 4 healthy volunteers and 6 patients. ns: not significant. ^∗^*P* < 0.05, ^∗∗^*P* < 0.01, and ^∗∗∗^*P* < 0.001.

BM-MSC possess a powerful immune regulatory function which has great potential in the treatment of autoimmune diseases including SLE. Although MSC can inhibit the pathological autoactivated lymphocytes, the underlying mechanism remains elusive. Considering the defective MEK/ERK signaling pathway of T cells from SLE patients, we hypothesized that MSC could activate the ERK pathway of PBMC from SLE patients. Interestingly, the phosphorylation of MEK (0.76 ± 0.25 vs. 1.18 ± 0.30, *P* = 0.04) and ERK (0.37 ± 0.19 vs. 1.27 ± 0.35, *P* = 0.0001) protein increased in PBMC on the third day when PBMC from SLE patients and BM-MSC from healthy volunteers were mixed cultured at a 15 : 1 ratio compared with the no-coculture group (Figures 2(a) and 2(c)). Despite the upregulation of phosphorylation of ERK in PBMC from healthy controls (0.32 ± 0.19 vs. 0.78 ± 0.35, *P* = 0.04), BM-MSC have no significant effect on the expression index of p-MEK in the same condition statistically (Figures 2(c) and 2(d)). In addition, the MEK/ERK signaling pathway was comparable in PBMC from SLE and HC subjects (Figures 2(a) and 2(c)). More importantly, the defection of the ERK pathway led to methylation alteration in T cells, which caused immune dysfunction in lupus. The DNA methyltransferases include DNMT1-2, 3A, 3B, and 3L isoforms. Among these DNA methyltransferases, DNMT1 that was regulated by the ERK pathway and related to the SLEDIA score gained our attention. We therefore evaluated the expression of DNMT1 of PBMC after the coculture with BM-MSC. Interestingly, both the mRNA and protein (0.40 ± 0.16 vs. 0.88 ± 0.27, *P* = 0.0098) levels of DNMT1 of SLE PBMC were significantly raised (Figures 2(e)–2(g)) after the coculture with BM-MSC for 4 days. In contrast, the healthy control group exhibited a slight increase in DNMT1 expression (0.53 ± 0.21 vs. 0.80 ± 0.27, *P* = 0.047).

### 3.3. BM-MSC Alter the Expression of Methylation-Sensitive Genes of SLE PBMC

**Figure 3:**
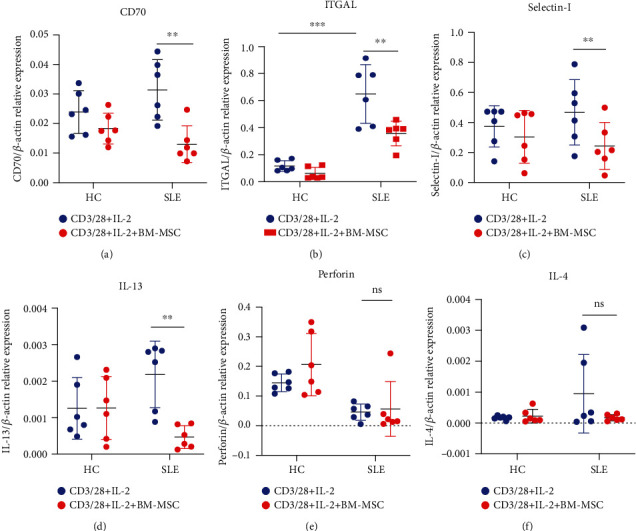
BM-MSC alter the expression of methylation-sensitive genes of SLE PBMC. PBMC were cultured in the same procedure as in Figure 2 for 5 days. The mRNA levels of methylation-sensitive genes including CD70, selectin-l, IL-13, ITGAL, perforin, and IL-4 of PBMC from healthy controls and SLE patients were measured by real-time PCR. The data are presented as the mean ± SD of 6 healthy volunteers and 6 patients. ^∗^*P* < 0.05, ^∗∗^*P* < 0.01, and ^∗∗∗^*P* < 0.001.

DNA methylation is indispensable to the epigenetic regulation of the target gene. The methylation of regulatory elements suppresses gene expression. Typically, hypomethylation is common in active genes. Additionally, early studies demonstrated that the inhibition of DNA methylation often caused aberrant expression of methylation-sensitive genes such as CD70 and ITGAL, which contributed to the autoactivation of T cells. Since we found that BM-MSC upregulated the expression of DNMT1, we assumed that BM-MSC could alter the expression of methylation-sensitive genes of PBMC from SLE patients. After the coculture with BM-MSC for 5 days, CD70, a costimulatory molecule of B cells, decreased in comparison with that in the control group (0.03 ± 0.01 vs. 0.01 ± 0.01, *P* = 0.002) (Figure 3(a)). However, the expression of CD70 in single-culture group was comparable to that in the coculture group of PBMC from healthy volunteers. Similar results could be observed in the expression of selectin-l (0.47 ± 0.22 vs. 0.24 ± 0.16, *P* = 0.0085) and IL-13 (0.0022 ± 0.0009 vs. 0.0005 ± 0.0003, *P* = 0.005) (Figures 3(b) and 3(d)), both of which increased in PBMC from SLE patients. Moreover, ITGAL (also known as CD11a or LFA-1), whose overexpression promoted T cell autoactivation, was downregulated after the coculture with BM-MSC (0.65 ± 0.22 vs. 0.36 ± 0.09, *P* = 0.002) (Figure 3(d)). The expressions of those genes of PBMC from SLE patients were also higher than those from the healthy controls (0.12 ± 0.04 vs. 0.65 ± 0.22, *P* < 0.001). In addition, perforin and IL-4 did not change after the coculture with MSC (Figures 3(e) and 3(f)). Taken together, we reveal the idea that BM-MSC may downregulate the expression of methylation-sensitive genes and then suppress the autoactivated PBMC via the MEK/ERK signaling pathway.

## 4. Discussion

BM-MSC can secrete regulatory factors and inhibit the excessive activation of SLE lymphocytes [20]. Clinically, the transplantation of bone marrow mesenchymal stem cells (BM-MSC) from healthy volunteers improves the disease activity and proteinuria [21]. However, the underlying mechanism is unclear. Emerging evidences indicated that the impaired extracellular ERK pathway of the CD4+ T cell subset contributed to the pathogenesis of SLE [22]. Indeed, hydralazine in concert with MEK inhibitors PD98059 or U0126 caused the drug-induced lupus [23]. Although previous studies had indicated that SLE could be ameliorated by normalizing the ERK signaling pathway [24], we for the first time found that MSC activated the MEK/ERK pathway of SLE PBMC. Because of the strong correlation between the ERK signaling pathway and SLE, we assumed that MSC improved SLE by increasing the ERK pathway of PBMC.

Next, we wanted to figure out how BM-MSC suppressed the autoactivation of PBMC from SLE patients after the normalization of the MEK/ERK signaling pathway. DNMT1 is a DNA methyltransferase that maintains the methylation pattern of target genes [14, 24]. The deficiency of DNMT1 will result in the progress of lupus [25, 26]. Lower expression of DNMT1 and hypomethylation of T cells could be observed in SLE patients [18] [27]. Furthermore, the expression of DNMT1 is regulated by the MEK/ERK signaling pathway [28]. We found that the PBMC from patients expressed more DNMT1 following the activation of the ERK pathway after the coculture with MSC. Therefore, we inferred that MSC might regulate the expression of DNMT1 by phosphorylating the MEK/ERK signaling pathway. However, unlike other studies, our research showed that the expression of DNMT1 of patients was similar to that of the healthy controls in our model. This may be due to the limited samples and the significant individual differences. Moreover, the epigenetic mechanism of lupus suggests that the abnormal DNA hypomethylation of T cells results in the excessive expression of methylation-sensitive autoimmunity-related genes such as CD70, ITGAL, selectin-l, IL-4, and IL-13 in lupus [29, 30]. CD70 is a costimulatory molecule expressed on T cells, and it can activate B cells [31]. ITGAL (CD11a) is part of the leukocyte function-associated antigen-1 (LFA-1, integrin *α*L*β*2, CD11a/CD18), which plays a central role in cellular adhesion and autoactivation of T cells. Selectin-l contributes to the formation of the steady immunological synapse, which exacerbates the activation of lymphocytes [32]. Furthermore, IL-4 and IL-13 are inflammatory factors taking part in the humoral immune response. Zhao et al.'s [33] and Zhang et al.'s [34] studies also reveal that targeting the DNA methyltransferase 1 can be a potential therapeutic approach to lupus. Similarly, our results revealed that in PBMC from SLE patients, several genes including CD70, ITGAL, selectin-l, and IL-13, all of which are hypomethylated and intensify lupus in different ways, are reduced by MSC. However, the expression of perforin and IL-4 changed little, probably because both of them can be regulated by other factors besides DNMT1. For example, Gaad45*α* may regulate perforin and IL-4 by decreasing global methylation [35]. What is more, DNA hydroxyl methylation, which is regulated by TET1, TET2, and 5hmC, also influences the level of perforin and IL-4 [22, 36].

Collectively, we showed for the first time that the MEK/ERK signaling pathway is activated and the expression of DNMT1 is upregulated in PBMC from SLE patients after the coculture with MSC from healthy volunteers. In the meantime, the pathological genes such as CD70, ITGAL, selectin-l, and IL-13 are downregulated. A comprehensive study about how MSC regulate the MEK/ERK signaling pathway and lead to the increase in DNMT1 is of interest and warrants further investigation. Thus, our findings indicate that BM-MSC improves SLE patients via the MEK/ERK-DNMT1 axis.

## Figures and Tables

**Table 1:** The primers of real-time PCR analysis.

Human *β*-actin	Fw: TCGCCAGCAACCTGAATCTC
	Rv: GCACGAAGCTCTTAGCGTCA
Human DNMT1	Fw: CCTAGCCCCAGGATTACAAGG
	Rv: ACTCATCCGATTTGGCTCTTTC
Human CD70	Fw: GCTTTGGTCCCATTGGTCG
	Rv: CGTCCCACCCAAGTGACTC
Human selectin-l	Fw: TGCCGAGACAATTACACAGATTT
	Rv: TGAAAGGCAGAGTCTTCTCCAG
Human ITGAL	Fw: TGCTTATCATCATCACGGATGG
	Rv: CTCTCCTTGGTCTGAAAATGCT
Human perforin	Fw: GTGGGACAATAACAACCCCAT
	Rv: TGGCATGATAGCGGAATTTTAGG
Human IL-4	Fw: ATGGGTCTCACCTCCCAACT
	Rv: GATGTCTGTTACGGTCAACTCG
Human IL-13	Fw: GAGGATGCTGAGCGGATTCTG
	Rv: CACCTCGATTTTGGTGTCTCG

## Data Availability

The data used to support the findings of this study are available from the corresponding author upon request.

## Supplementary Materials

Supplementary Materials Supplementary Figure 1: the mRNA level of DNMT1 of SLE PBMC was not significantly raised after the coculture with BM-MSC for 3 days (0.1822 ± 0.095 vs. 0.2694 ± 0.1487, *P* = 0.5637). Supplementary Figure 2: (A–C) the levels of CD70 (*P* = 0.0806), ITGAL (*P* = 0.3088), and selectin-l (*P* = 0.6946) changed little after the coculture with BM-MSC for 4 days. (D) We can only observe that the mRNA level of IL-13 decreased in comparison with that in the control group (0.0020 ± 0.0015 vs. 0.0005 ± 0.0005, *P* = 0.0316) on the 4th coculture day. Supplementary Figure 3: the living cell ratio of PBMC from both control and coculture groups reaches more than 90%. Supplementary Figure 4: the PBMC from SLE patients expressed lower IL-2 than that from healthy people (0.0006 ± 0.0006 vs. 0.0038 ± 0.0022, *P* = 0.0056). (A) However, it seems that BM-MSC impacts poorly (*P* = 0.9101) on the level of IL-2. It may be because the DNA methylation of IL-2 is regulated by other molecules. What is more, IL-2 added in the medium can also influence the mRNA level of PBMC. (B) And the level of IFN-*γ* also changed little after being cocultured with BM-MSC.
