# Supplementary material for: Mesenchymal Stem Cells Activate the MEK/ERK Signaling Pathway and Enhance DNA Methylation via DNMT1 in PBMC from Systemic Lupus Erythematosus
Source: Biomed Res Int. 2020 Nov 17;2020:4174082. doi: 10.1155/2020/4174082 (PMC7685810; doi:10.1155/2020/4174082)
Supplement: Supplementary Materials — Supplementary Figure 1: the mRNA level of DNMT1 of SLE PBMC was not significantly raised after the coculture with BM-MSC for 3 days (0.1822 ± 0.095 vs. 0.2694 ± 0.1487, P = 0.5637). Supplementary Figure 2: (A–C) the levels of CD70 (P = 0.0806), ITGAL (P = 0.3088), and selectin-l (P = 0.6946) changed little after the coculture with BM-MSC for 4 days. (D) We can only observe that the mRNA level of IL-13 decreased in comparison with that in the control group (0.0020 ± 0.0015 vs. 0.0005 ± 0.0005, P = 0.0316) on the 4th coculture day. Supplementary Figure 3: the living cell ratio of PBMC from both control and coculture groups reaches more than 90%. Supplementary Figure 4: the PBMC from SLE patients expressed lower IL-2 than that from healthy people (0.0006 ± 0.0006 vs. 0.0038 ± 0.0022, P = 0.0056). (A) However, it seems that BM-MSC impacts poorly (P = 0.9101) on the level of IL-2. It may be because the DNA methylation of IL-2 is regulated by other molecules. What is more, IL-2 added in the medium can also influence the mRNA level of PBMC. (B) And the level of IFN-γ also changed little after being cocultured with BM-MSC. [file 4174082.f1.docx]

**Supplementary Fig.1**.The mRNA of DNMT1 of PBMC were determined by real-time PCR after 3 days. The data are presented as the mean ± SD of 4 healthy volunteers and 6 patients. (ns, not significant, * p < 0.05, ** p < 0.01 and *** p < 0.001)

**Supplementary Fig.2**. PBMC were cultured in the same procedure as in Fig. 3 for 4 days. The mRNA levels of methylation-sensitive genes including CD70, selectin-l, IL-13, ITGAL, perforin and IL-4 of PBMC from healthy controls and SLE patients were measured by real time PCR. The data are presented as the mean ± SD of 6 healthy volunteers and 6 patients. (ns, not significant, * p < 0.05, ** p < 0.01 and *** p < 0.001)

**Supplementary Fig.3**. PBMC were stained with FITC conjugated anti-CD45 ( #555482) and APC-Cy7 conjugated Fixable Viability Stain 780 ( #565388) (Becton Dickinson, USA) after cultured for 5 days. Then PBMC were analyzed by a Flow Cytometer (BD FACSVere, USA). PBMC were positive for CD45 and the live cell subset had weaker fluorescence intensity in APC-Cy7. (A) The living cell ratio of PBMC in control group is 97.9%. (B) The living cell ratio in co-culture group is 95.4%.

**Supplementary Fig.4**. PBMC were cultured in the same procedure for 5 days. The mRNA levels of IL-2、IFN-γ of PBMC from healthy controls and SLE patients were measured by real time PCR. The data are presented as the mean ± SD of 6 healthy volunteers and 6 patients. (ns, not significant, * p < 0.05, ** p < 0.01 and *** p < 0.001)
